# Synthesis and crystal structure of a solvated Co^III^ complex with 2-hy­droxy-3-meth­oxy­benzaldehyde thio­semicarbazone ligands

**DOI:** 10.1107/S2056989021010616

**Published:** 2021-10-21

**Authors:** Julia A. Rusanova, Volodymyr N. Kokozay, Svitlana Petrusenko, Nataliya Plyuta

**Affiliations:** a Department of Chemistry, Taras Shevchenko National University of Kyiv, 64/13, Volodymyrska str., Kyiv 01601, Ukraine

**Keywords:** crystal structure, Co^III^, 2-hy­droxy-3-meth­oxy­benzaldehyde thio­semicarbazone

## Abstract

The title Co^III^ complex, bis­[bis­(2-hy­droxy-3-meth­oxy­benzaldehyde thio­semi­carbazonato)cobalt(III)] di­thio­nate–dimethylformamide–methanol (1/4/3), [Co(C_9_H_10_N_3_O_2_S)_2_]_2_(S_2_O_6_)·4C_3_H_7_NO·3CH_3_OH, with monodeprotonated 2-hy­droxy-3-meth­oxy­benzaldehyde thio­semicarbazone as ligands crystallizes in the space group *P*  . The asymmetric unit consists of two mononuclear [Co*L*_2_]^+^ cations, one di­thio­nate anion (S_2_O_6_)^2−^ as counter-anion and seven solvate mol­ecules (four di­methyl­methanamide and three methanol). Each Co^III^ ion has a moderately distorted octa­hedral S_2_N_2_O_2_ geometry. In the crystal, the components are linked by numerous N—H⋯O and O—H⋯O contacts.

## Abstract 2

The synthesis, crystal structure and spectroscopic characterization of the novel and, according to our knowledge the first to be obtained in crystalline form, Co^III^ complex with a multidentate NSO-containing mixed-ligand − 2-hy­droxy-3-meth­oxy­benzaldehyde thio­semicarbazone – is reported.

## Chemical context

In recent years, Schiff bases have played a vital role in the progress of modern coordination chemistry, in the improvement of the areas of magnetism, luminescence, chirality, catalysis, cytotoxicity and ferroelectricity (Andruh *et al.*, 2015 ▸; Mishra *et al.*, 2016 ▸; Aazam & El-Said, 2014 ▸). Thio­semicarbazones represent an important class of Schiff base sulfur-donor ligands, particularly for many transition-metal ions. These metal complexes have received considerable attention, primarily because of their bioinorganic relevance (Gupta *et al.*, 2003 ▸ *;* Singh *et al.*, 2000 ▸ *):* they are promising drug candidates, biomarkers and biocatalysts (Hayne *et al.*, 2014 ▸ *;* Lim *et al.*, 2010 ▸). It has been noted that some metal(II) complexes with thio­semicarbazone-derived ligands have the ability to induce apoptosis in cancerous cell lines (Ferrari *et al.*, 2004 ▸; Santini *et al.*, 2014 ▸). 
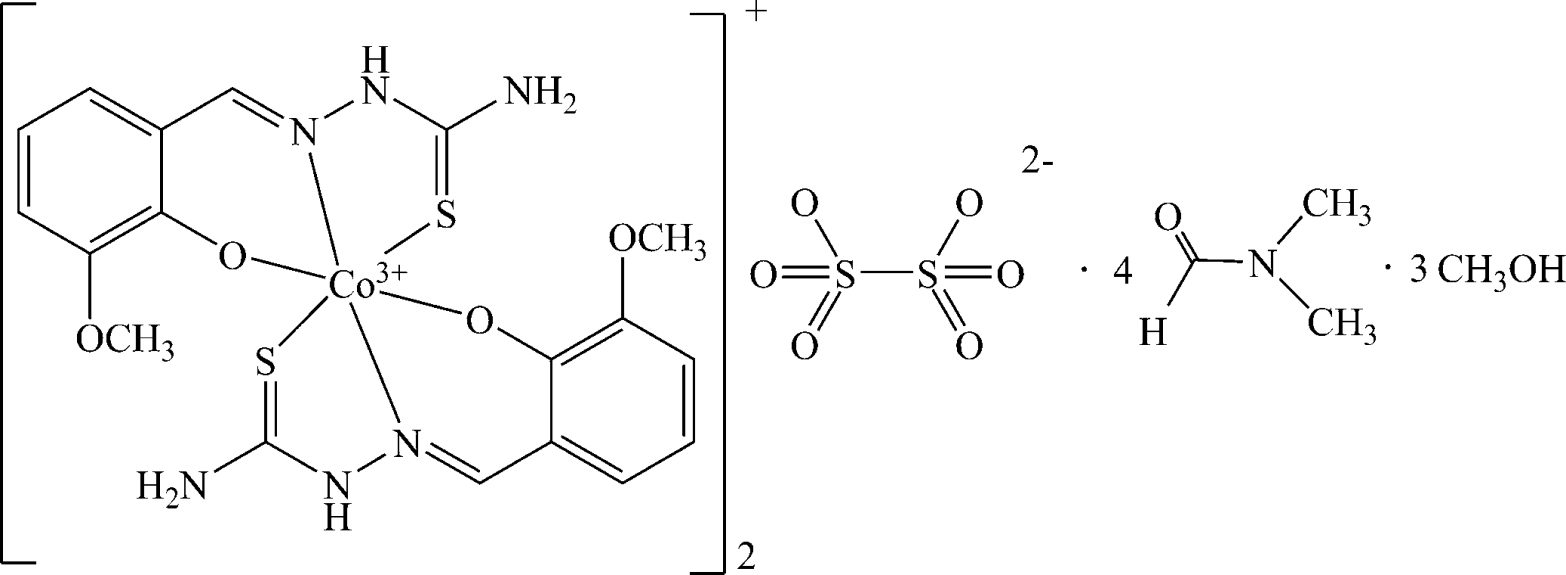


Despite the attention towards Schiff bases, thio­semi­carbazones and their metal complexes, very few studies have been devoted to the synthesis and crystal-structure determinations of Co complexes. In this work, we present the synthesis, crystal structure and spectroscopic characterization of the novel and, according to our knowledge, the first to be obtained in crystalline, form Co^III^ complex with the multidentate NSO-containing mixed-ligand 2-hy­droxy-3-meth­oxy­benzaldehyde thio­semicarbazone.

## Structural commentary

**Figure 1:**
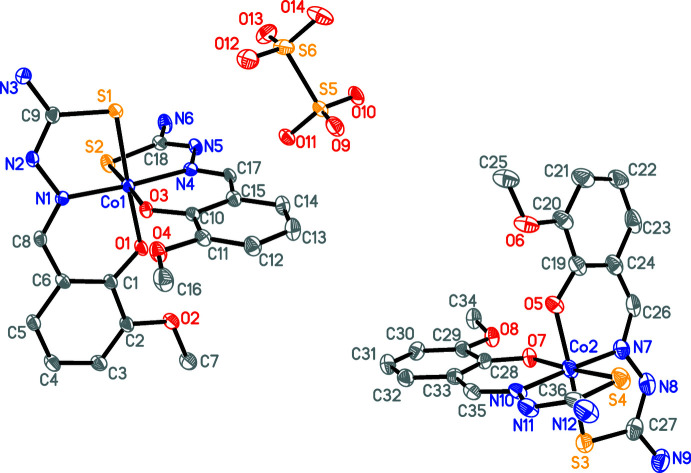
The mol­ecular structure of the title compound with the atom-labeling scheme. Displacement ellipsoids are drawn at the 30% probability level. Solvent mol­ecules (di­methyl­formamide and methanol) are omitted for clarity.

The title complex crystallizes in the triclinic space group *P* 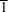 . The asymmetric unit (Fig. 1 ▸) consists of two independent mononuclear complex cations, a di­thio­nate anion as counter-anion and seven solvent mol­ecules of crystallization (four di­methyl­methanamide and three methanol). Each Co^III^ ion is coordinated by two monodeprotonated (by the phenol group) ONS tridentate thio­semicarbazone ligands through the phenoxo oxygen, imine nitro­gen and thione sulfur atoms. Thus, the coordination geometry around each Co^III^ ion can be described as moderately distorted octa­hedral with an S_2_N_2_O_2_ coordination sphere with N,O,N and S atoms in the equatorial plane and O and S atoms in the apical positions.

In the title compound, the two Co—N, Co—O and Co—S distances are each almost identical (the mean values being 1.89, 1.92 and 2.22 Å, respectively) to those in an analogous chromium complex with a similar ligand (CCDC refcode YIMPER; Chumakov *et al.*, 2013 ▸). At the same time, the Co—O and Co—N distances in the title complex are shorter than in analogous Co^II^ complexes with related semicarbazone ligands (Co—N = 2.041 Å and Co—O = 2.056 Å in VAYZUT, VAYZON and VAZBAC; Wu *et al.*, 2017 ▸). The Co—S distances in the title complex are in the range 2.2202 (19)-2.2269 (17) Å, which is generally comparable to the range 2.23–2.24 Å observed for a Co^III^ complex (VENDIB; Burstein *et al.*, 1988 ▸) and shorter than was found for the Co^II^ complex of glyoxylic acid with thio­semicarbazone (2.419–2.424 Å; ODOWUC; Huseynova *et al.*, 2018 ▸).

Despite the ligands coordinating to the Co^III^ cations through the thione sulfur atoms, the C—S bond length of the thio­semicarbazone moiety (average length of 1.71 Å) approaches the standard C=S double-bond value and differs only slightly from the distance observed in the corresponding neutral ligand [1.688 Å in BIZYAL (Zhao *et al.*, 2008 ▸) and 1.697 Å in BIZYAL01 (Vrdoljak *et al.*, 2010 ▸)].

The ligands coordinated to the Co^III^ ions are almost planar (r.m.s. deviations of fitted atoms are 0.0793 and 0.0917 Å for the ligands coordinated to Co1 and 0.0862 and 0.0785 Å for the ligands coordinated to Co2) and twisted, as defined by the dihedral angles of 83.42 (7)° between the mean planes of atoms O1/C1/C6/C8/N1/N2/C9/S1 and O3/C10/C15/C17/N4/N5/C18/S2 around Co1, and 86.3 (1)° between the mean planes of atoms O7/C28/C33/C35/N10/N11/C36/S4 and O5/C19/C24/C26/N10/N8/C27/S3 around Co2.

## Supra­molecular features

**Figure 2:**
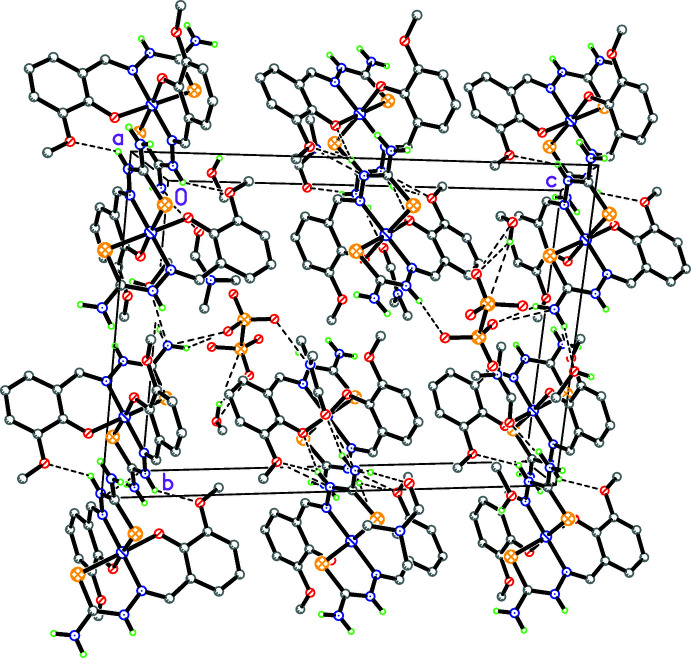
The crystal packing of the title compound viewed along the *a* axis. N—H⋯O and O—H⋯O hydrogen bonds, which link the components in the crystal, are shown as dashed lines. C-bound hydrogen atoms are omitted for clarity.

**Figure 3:**
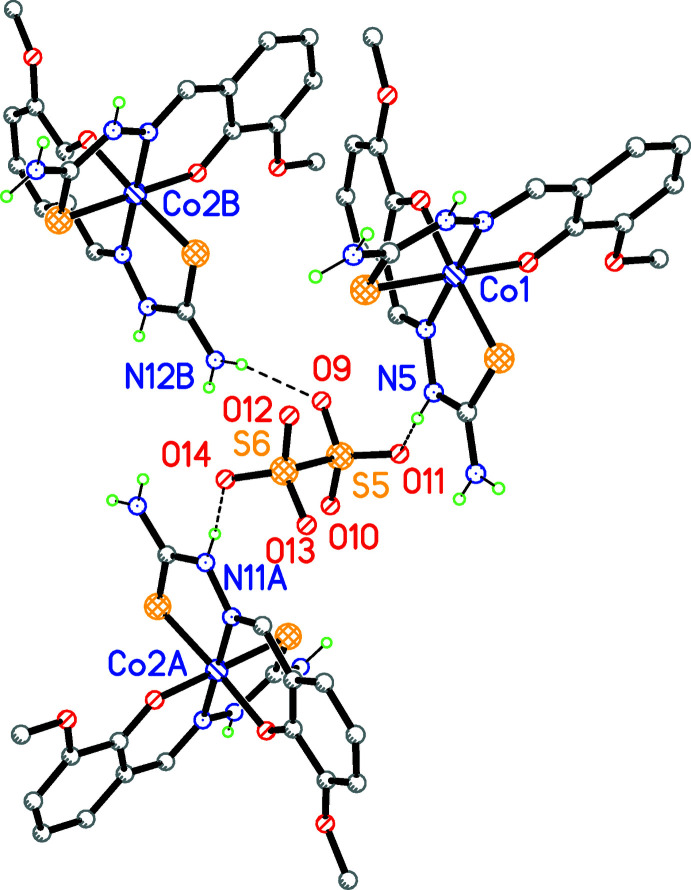
A fragment of the packing of the title compound demonstrating the N–H⋯O contacts that link three complex cations and an S_2_O_6_^2−^ anion as an hydrogen-bond acceptor. Hydrogen bonds are shown as dashed lines. Methanol solvate mol­ecules bonded to S_2_O_6_^2−^ by O—H⋯O hydrogen bonds, dimethyformamide solvent mol­ecules and C-bound hydrogen atoms are omitted for clarity.

The solid-state organization of the complex can be described as an insertion of the anions and solvent mol­ecules within the crystallographically independent complexes (Fig. 2 ▸). In the crystal, the components are linked by numerous N–H⋯O and O–H⋯O contacts (Table 1 ▸), giving a three-dimensional hydrogen-bonded network. Overall, the amino groups of the coordinated ligands are involved in eleven N—H⋯O contacts:

N8—H8*A*⋯O8, N2—H2⋯O3 and N2—H2⋯O4 are contacts between ligands through the nitro­gen of the secondary amino group and meth­oxy group oxygen;

N11—H11⋯O14, N5—H5*A*⋯O11 and N12—H12*B*⋯O9 are contacts between the nitro­gen of the secondary and primary (terminal) amino groups of the ligands and oxygen atoms of the S_2_O_6_ anions (Fig. 3 ▸);

N3—H3*B*⋯O17, N3—H3*A*⋯O15, N9—H9*A*⋯O18, N6—H6*B*⋯O16, N9—H9*B*⋯O19 and N12—H12*A*⋯O21*A* are contacts between nitro­gen of the primary amino groups of the ligands and the oxygen atoms of solvent mol­ecules (O15, O16, O17, O18 of di­methyl­methanamide and O19, O21 of methanol).

**Figure 4:**
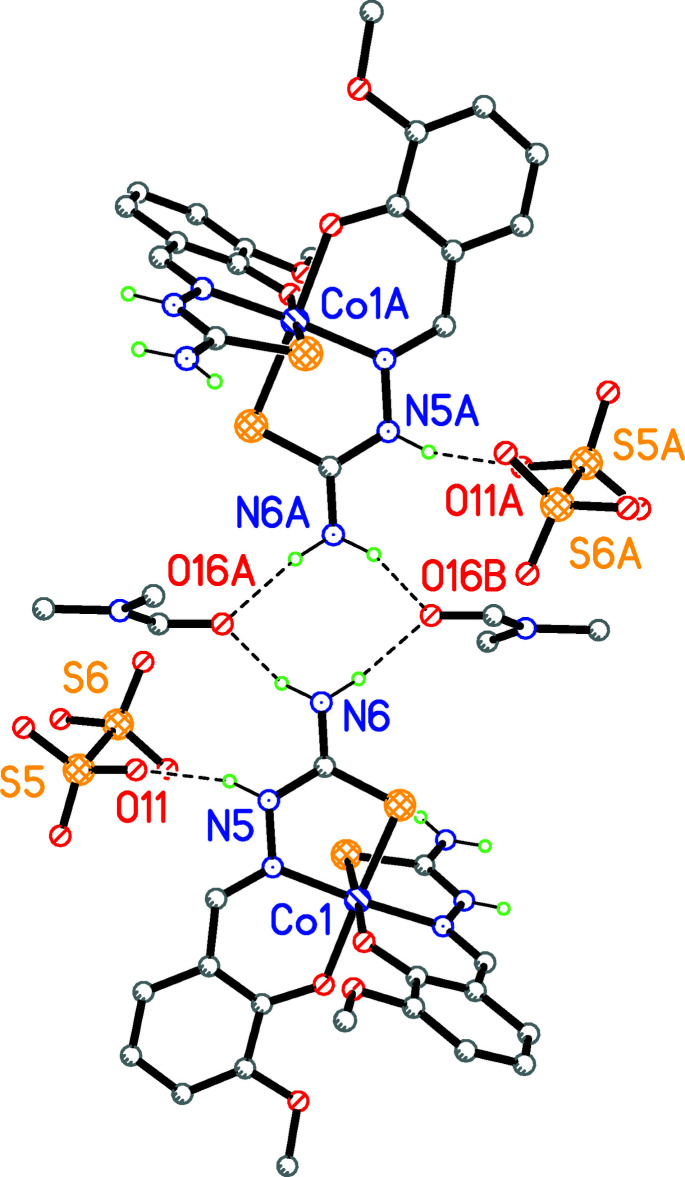
A fragment of the crystal packing of the title compound showing the double NH_2_⋯O(DMF)⋯H_2_N contacts that link the complex cations with two dimethyformamide mol­ecules through bridging oxygen atoms. C-bound hydrogen atoms and rest of the solvent mol­ecules are omitted for clarity.

The (S_2_O_6_)^2−^ anions act as a multiple-acceptor species for N,O donor atoms of neighboring complexes (by N—H⋯O inter­actions) and methanol solvent mol­ecules (by O—H⋯O contacts). The oxygen atoms (O16) of the di­methyl­methanamide mol­ecules bridge adjacent cationic complexes (Fig. 4 ▸).

## Database survey

A search of the Cambridge Structural Database (Version 5.42; last update November 2020; Groom *et al.*, 2016 ▸) for related transition-metal complexes with 2-hy­droxy-3-meth­oxy­benzaldehyde thio­semicarbazone gave 33 hits and only two hits for Co complexes with thio­semicarbazones, *viz*. ODOWUC (Huseynova *et al.*, 2018 ▸) and VENDIB (Burstein, *et al.*, 1988 ▸).

## Synthesis and crystallization

The title compound was prepared according to a previously published procedure (Rusanov *et al.*, 2003 ▸) by slow inter­diffusion of a solution of 0.086 g (0.26 mmol) of CoS_2_O_6_·6H_2_O in 1ml of methanol and 0.117g (0.52 mmol) of the ligand in 1ml of di­methyl­formamide and 1ml of chloro­form. Dark-brown crystals of the title compound, suitable for X-ray analysis, were formed within a few days (yield: 60%).

The IR spectrum of the title compound (as KBr pellets) is consistent with the above structural data. In the range 4000–400 cm^−1^ it shows all characteristic peaks: υ(CH) due to aromatic =C—H stretching at 3000–3100 cm^−1^, the aromatic ring vibrations in the 1600–1400 cm^−1^ region, weak absorption band at 738 cm^−1^ due to υ(C—S) vibrations and the characteristic peak at 1608 cm^−1^ assigned to azomethine υ(C=N) group. The weak band at 3308 cm^−1^ can be assigned to the N—H group vibrations. All these data are in good agreement with literature data (Seena & Kurup, 2007 ▸; Kalaivany *et al.*, 2014 ▸). Analysis calculated for C_51_H_80_Co_2_N_16_O_21_S_6_ (*M* = 1563.53): C 38.19; N 14.33; H 5.16%. Found: C 38.21; N 14.40; H 5.21%.

The Co^II^ di­thio­nate used in this work was prepared by mixing aqueous solutions containing stoichiometric amounts of cobalt sulfate and BaS_2_O_6_·2H_2_O. The white precipitate of BaSO_4_ was removed by filtration and the solution containing the metal di­thio­nate was evaporated to a small volume on a rotary evaporator and then cooled for crystallization. BaS_2_O_6_·2H_2_O was prepared using the method described by Pfanstiel (1946 ▸).

## Refinement

Crystal data, data collection and structure refinement details are summarized in Table 2 ▸. All non-hydrogen atoms were refined anisotropically. One of the methanol mol­ecules is disordered over two positions with relative occupancies of 0.597 (17) and 0.403 (17) for the major and minor components. The hydrogen atoms bonded to carbon were included at geometrically calculated positions and as riding with *U*_iso_(H) = 1.2*U*_eq_(C) for aromatic CH and *U*_iso_(H) = 1.5*U*_eq_(C) for methyl groups. The H atoms of the NH and OH groups were also placed at calculated position using the corresponding AFIX instruction with *U*_iso_(H) = 1.2*U*_eq_(N) for NH/NH_2_ and *U*_iso_(H) = 1.5*U*_eq_(O) for OH hydrogen atoms.

## Supplementary Material

Crystal structure: contains datablock(s) I. DOI: 10.1107/S2056989021010616/tx2043sup1.cif

Structure factors: contains datablock(s) I. DOI: 10.1107/S2056989021010616/tx2043Isup2.hkl

CCDC reference: 2115486

Additional supporting information:  crystallographic information; 3D view; checkCIF report

## Figures and Tables

**Table 1:** Hydrogen-bond geometry (Å, °)

*D*—H⋯*A*	*D*—H	H⋯*A*	*D*⋯*A*	*D*—H⋯*A*
N8—H8*A*⋯O8^i^	0.88	2.28	2.969 (7)	135
N2—H2⋯O3^ii^	0.88	2.27	2.999 (5)	140
N2—H2⋯O4^ii^	0.88	2.01	2.740 (6)	140
N11—H11⋯O14^iii^	0.88	2.02	2.877 (7)	165
N5—H5*A*⋯O11	0.88	1.98	2.813 (6)	157
N12—H12*B*⋯O9^iv^	0.88	2.12	2.911 (7)	150
N3—H3*B*⋯O17^v^	0.88	1.98	2.839 (8)	166
N3—H3*A*⋯O15^ii^	0.88	2.05	2.881 (7)	156
N9—H9*A*⋯O18^i^	0.88	1.89	2.756 (8)	168
N6—H6*B*⋯O16^vi^	0.88	1.95	2.822 (6)	169
N9—H9*B*⋯O19^vii^	0.88	1.97	2.834 (9)	165
N12—H12*A*⋯O21*A* ^iv^	0.88	2.06	2.878 (12)	155
O19—H19⋯O20	0.84	1.90	2.720 (9)	167
O20—H20⋯O12	0.84	2.01	2.722 (8)	142

Symmetry codes: (i) -x+1, -y, -z; (ii) -x+1, -y+2, -z+1; (iii) x+1, y, z; (iv) -x+1, -y+1, -z; (v) x-1, y, z; (vi) -x+1, -y+1, -z+1; (vii) x+1, y-1, z.

**Table 2:** Experimental details

Crystal data	
Chemical formula	[Co(C_9_H_10_N_3_O_2_S)_2_]_2_(S_2_O_6_)·4C_3_H_7_NO·3CH_4_O
*M*_r_	1563.53
Crystal system, space group	Triclinic, *P*\overline{1}
Temperature (K)	133
*a*, *b*, *c* (Å)	13.0652 (8), 14.1171 (9), 19.9233 (12)
α, β, γ (°)	93.179 (2), 106.381 (2), 99.884 (2)
*V* (Å^3^)	3452.2 (4)
*Z*	2
Radiation type	Mo *K*α
μ (mm^−1^)	0.74
Crystal size (mm)	0.46 × 0.14 × 0.05

Data collection	
Diffractometer	Bruker APEXII CCD
Absorption correction	Multi-scan (*SADABS*; Krause *et al.*, 2015 ▸)
*T*_min_, *T*_max_	0.632, 0.745
No. of measured, independent and observed [*I* > 2σ(*I*)] reflections	51207, 12244, 8043
*R*_int_	0.077
(sin θ/λ)_max_ (Å^−1^)	0.596

Refinement	
*R*[*F* ^2^ > 2σ(*F* ^2^)], *wR*(*F* ^2^), *S*	0.071, 0.203, 1.02
No. of reflections	12244
No. of parameters	896
No. of restraints	7
H-atom treatment	H-atom parameters constrained
Δρ_max_, Δρ_min_ (e Å^−3^)	1.08, −0.66

Computer programs: *APEX2* and *SAINT* (Bruker, 2008 ▸), *SHELXT* (Sheldrick, 2015*a* ▸), *SHELXL2016/4* (Sheldrick, 2015*b* ▸), *SHELXTL* (Sheldrick, 2008 ▸) and *publCIF* (Westrip, 2010 ▸).

## supplementary crystallographic information

### Crystal data

**Table:**

[Co(C_9_H_10_N_3_O_2_S)_2_]_2_(S_2_O_6_)·4C_3_H_7_NO·3CH_4_O	*Z* = 2
*M**_r_* = 1563.53	*F*(000) = 1632
Triclinic, *P*1	*D*_x_ = 1.504 Mg m^−^^3^
*a* = 13.0652 (8) Å	Mo *K*α radiation, λ = 0.71073 Å
*b* = 14.1171 (9) Å	Cell parameters from 6474 reflections
*c* = 19.9233 (12) Å	θ = 2.3–23.7°
α = 93.179 (2)°	µ = 0.74 mm^−^^1^
β = 106.381 (2)°	*T* = 133 K
γ = 99.884 (2)°	Plate, brown
*V* = 3452.2 (4) Å^3^	0.46 × 0.14 × 0.05 mm

### Data collection

**Table:**

Bruker APEXII CCD diffractometer	8043 reflections with *I* > 2σ(*I*)
Radiation source: sealed tube	*R*_int_ = 0.077
φ and ω scans	θ_max_ = 25.1°, θ_min_ = 1.1°
Absorption correction: multi-scan (SADABS; Krause *et al.*, 2015)	*h* = −15→15
*T*_min_ = 0.632, *T*_max_ = 0.745	*k* = −16→16
51207 measured reflections	*l* = −23→23
12244 independent reflections	

### Refinement

**Table:**

Refinement on *F*^2^	7 restraints
Least-squares matrix: full	Hydrogen site location: inferred from neighbouring sites
*R*[*F*^2^ > 2σ(*F*^2^)] = 0.071	H-atom parameters constrained
*wR*(*F*^2^) = 0.203	*w* = 1/[σ^2^(*F*_o_^2^) + (0.0952*P*)^2^ + 6.9557*P*] where *P* = (*F*_o_^2^ + 2*F*_c_^2^)/3
*S* = 1.02	(Δ/σ)_max_ = 0.011
12244 reflections	Δρ_max_ = 1.08 e Å^−^^3^
896 parameters	Δρ_min_ = −0.66 e Å^−^^3^

### Special details

**Table:**

Geometry. All esds (except the esd in the dihedral angle between two l.s. planes) are estimated using the full covariance matrix. The cell esds are taken into account individually in the estimation of esds in distances, angles and torsion angles; correlations between esds in cell parameters are only used when they are defined by crystal symmetry. An approximate (isotropic) treatment of cell esds is used for estimating esds involving l.s. planes.

### Fractional atomic coordinates and isotropic or equivalent isotropic displacement parameters (Å^2^)

**Table:**

	*x*	*y*	*z*	*U*_iso_*/*U*_eq_	Occ. (<1)
CO1	0.36292 (5)	0.79760 (5)	0.45380 (4)	0.02529 (19)	
CO2	0.66130 (7)	0.20873 (6)	0.02449 (4)	0.0409 (2)	
S1	0.24082 (11)	0.88224 (10)	0.40004 (7)	0.0314 (3)	
S2	0.25632 (11)	0.73953 (10)	0.51759 (7)	0.0341 (3)	
S3	0.80002 (14)	0.13375 (13)	0.06047 (9)	0.0506 (4)	
S4	0.72076 (14)	0.26612 (14)	−0.06245 (8)	0.0528 (5)	
S5	0.07958 (12)	0.47129 (12)	0.21986 (8)	0.0398 (4)	
S6	−0.02347 (13)	0.57359 (12)	0.21385 (10)	0.0494 (4)	
N1	0.4248 (3)	0.9061 (3)	0.5223 (2)	0.0240 (9)	
N2	0.3674 (3)	0.9807 (3)	0.5189 (2)	0.0297 (10)	
H2	0.390135	1.030246	0.551679	0.036*	
N3	0.2201 (4)	1.0455 (4)	0.4645 (2)	0.0407 (12)	
H3A	0.241240	1.093223	0.498756	0.049*	
H3B	0.160632	1.043479	0.429546	0.049*	
N4	0.2907 (3)	0.6901 (3)	0.3861 (2)	0.0258 (10)	
N5	0.1999 (3)	0.6318 (3)	0.3964 (2)	0.0290 (10)	
H5A	0.159020	0.585297	0.363801	0.035*	
N6	0.0909 (4)	0.5908 (3)	0.4661 (2)	0.0394 (12)	
H6A	0.051200	0.543641	0.433710	0.047*	
H6B	0.074240	0.600513	0.505412	0.047*	
N7	0.5832 (4)	0.0923 (4)	−0.0333 (2)	0.0439 (13)	
N8	0.6391 (5)	0.0187 (4)	−0.0361 (3)	0.0542 (15)	
H8A	0.607307	−0.035171	−0.064020	0.065*	
N9	0.7951 (5)	−0.0403 (4)	−0.0014 (3)	0.0645 (17)	
H9A	0.762313	−0.092407	−0.030988	0.077*	
H9B	0.863082	−0.035064	0.024444	0.077*	
N10	0.7434 (4)	0.3243 (4)	0.0811 (2)	0.0358 (11)	
N11	0.8075 (4)	0.3890 (4)	0.0522 (3)	0.0461 (13)	
H11	0.853189	0.439635	0.078298	0.055*	
N12	0.8491 (4)	0.4405 (5)	−0.0457 (3)	0.0621 (17)	
H12A	0.887922	0.494462	−0.020201	0.075*	
H12B	0.844118	0.431375	−0.090633	0.075*	
N13	0.6870 (5)	0.6158 (5)	0.4054 (3)	0.0684 (18)	
N14	0.9538 (4)	0.2600 (4)	0.3482 (3)	0.0477 (13)	
N15	0.9533 (6)	0.8744 (6)	0.3513 (4)	0.093 (2)	
N16	0.3835 (5)	0.3449 (5)	0.1472 (3)	0.0570 (15)	
O1	0.4674 (3)	0.7212 (2)	0.49697 (18)	0.0310 (8)	
O2	0.5854 (3)	0.5945 (3)	0.5397 (2)	0.0443 (11)	
O3	0.4626 (3)	0.8491 (2)	0.40128 (17)	0.0265 (8)	
O4	0.6132 (3)	0.9329 (3)	0.3504 (2)	0.0391 (10)	
O5	0.5439 (3)	0.2761 (3)	−0.0030 (2)	0.0408 (10)	
O6	0.4170 (3)	0.3980 (3)	−0.0349 (2)	0.0541 (12)	
O7	0.6011 (3)	0.1588 (3)	0.09775 (19)	0.0404 (10)	
O8	0.4994 (3)	0.0714 (3)	0.1780 (2)	0.0427 (10)	
O9	0.1597 (3)	0.5116 (4)	0.1872 (2)	0.0555 (12)	
O10	0.0089 (3)	0.3821 (3)	0.1837 (2)	0.0507 (11)	
O11	0.1252 (3)	0.4690 (3)	0.2951 (2)	0.0398 (10)	
O12	0.0495 (4)	0.6657 (3)	0.2445 (3)	0.0614 (13)	
O13	−0.0965 (4)	0.5396 (3)	0.2531 (3)	0.0623 (13)	
O14	−0.0773 (4)	0.5671 (3)	0.1380 (3)	0.0672 (15)	
O15	0.7544 (4)	0.7731 (4)	0.4530 (3)	0.0691 (15)	
O16	0.9759 (4)	0.4050 (3)	0.4111 (2)	0.0515 (12)	
O17	1.0408 (5)	1.0151 (5)	0.3403 (4)	0.112 (3)	
O18	0.3242 (4)	0.1861 (4)	0.1059 (3)	0.0703 (15)	
C1	0.5495 (4)	0.7517 (4)	0.5534 (3)	0.0287 (12)	
C2	0.6197 (5)	0.6846 (4)	0.5779 (3)	0.0348 (13)	
C3	0.7122 (5)	0.7107 (4)	0.6340 (3)	0.0371 (14)	
H3	0.758027	0.665433	0.648316	0.045*	
C4	0.7392 (5)	0.8034 (4)	0.6703 (3)	0.0385 (14)	
H4	0.803681	0.821182	0.708785	0.046*	
C5	0.6729 (4)	0.8686 (4)	0.6503 (3)	0.0316 (12)	
H5	0.690778	0.930985	0.675973	0.038*	
C6	0.5771 (4)	0.8439 (4)	0.5914 (3)	0.0285 (12)	
C7	0.6537 (5)	0.5261 (4)	0.5604 (4)	0.0503 (17)	
H7A	0.724650	0.549612	0.553484	0.075*	
H7B	0.619995	0.463904	0.531800	0.075*	
H7C	0.663363	0.517879	0.610183	0.075*	
C8	0.5115 (4)	0.9156 (4)	0.5748 (3)	0.0267 (12)	
H8	0.533624	0.974795	0.604773	0.032*	
C9	0.2776 (4)	0.9767 (4)	0.4656 (3)	0.0314 (12)	
C10	0.4698 (4)	0.8044 (4)	0.3434 (3)	0.0273 (12)	
C11	0.5519 (4)	0.8451 (4)	0.3137 (3)	0.0322 (13)	
C12	0.5678 (5)	0.8018 (4)	0.2549 (3)	0.0395 (14)	
H12	0.624872	0.831031	0.237440	0.047*	
C13	0.4999 (5)	0.7150 (4)	0.2209 (3)	0.0447 (15)	
H13	0.510878	0.684579	0.180425	0.054*	
C14	0.4173 (5)	0.6737 (4)	0.2460 (3)	0.0390 (14)	
H14	0.369851	0.615472	0.222075	0.047*	
C15	0.4022 (4)	0.7172 (4)	0.3073 (3)	0.0299 (12)	
C16	0.7002 (5)	0.9797 (5)	0.3260 (3)	0.0508 (17)	
H16A	0.752327	0.936971	0.327502	0.076*	
H16B	0.736876	1.040078	0.356249	0.076*	
H16C	0.670712	0.993965	0.277501	0.076*	
C17	0.3140 (4)	0.6663 (4)	0.3287 (3)	0.0286 (12)	
H17	0.269026	0.610955	0.298801	0.034*	
C18	0.1763 (4)	0.6476 (4)	0.4556 (3)	0.0291 (12)	
C19	0.4478 (5)	0.2412 (5)	−0.0481 (3)	0.0450 (16)	
C20	0.3731 (5)	0.3045 (5)	−0.0657 (3)	0.0471 (16)	
C21	0.2696 (5)	0.2727 (6)	−0.1089 (4)	0.0584 (19)	
H21	0.221108	0.316580	−0.119374	0.070*	
C22	0.2344 (5)	0.1760 (6)	−0.1380 (4)	0.058 (2)	
H22	0.162061	0.154136	−0.167622	0.070*	
C23	0.3036 (6)	0.1136 (6)	−0.1239 (3)	0.059 (2)	
H23	0.279187	0.048305	−0.144347	0.070*	
C24	0.4119 (5)	0.1435 (5)	−0.0791 (3)	0.0476 (17)	
C25	0.3450 (5)	0.4635 (5)	−0.0450 (4)	0.063 (2)	
H25A	0.316514	0.469071	−0.095398	0.094*	
H25B	0.383931	0.527113	−0.020252	0.094*	
H25C	0.284607	0.439863	−0.026392	0.094*	
C26	0.4791 (6)	0.0752 (5)	−0.0702 (3)	0.0481 (17)	
H26	0.447568	0.011354	−0.092357	0.058*	
C27	0.7424 (6)	0.0299 (5)	0.0043 (4)	0.0540 (18)	
C28	0.6151 (5)	0.2070 (4)	0.1586 (3)	0.0356 (13)	
C29	0.5593 (5)	0.1622 (4)	0.2048 (3)	0.0386 (14)	
C30	0.5682 (5)	0.2089 (4)	0.2694 (3)	0.0419 (15)	
H30	0.530361	0.177577	0.298931	0.050*	
C31	0.6312 (5)	0.3003 (5)	0.2920 (3)	0.0471 (16)	
H31	0.635118	0.332121	0.336232	0.057*	
C32	0.6883 (5)	0.3454 (5)	0.2502 (3)	0.0425 (15)	
H32	0.732835	0.407641	0.266326	0.051*	
C33	0.6813 (5)	0.3000 (4)	0.1836 (3)	0.0367 (14)	
C34	0.4267 (6)	0.0293 (5)	0.2142 (3)	0.0508 (17)	
H34A	0.467611	0.024454	0.262965	0.076*	
H34B	0.389621	−0.035395	0.190836	0.076*	
H34C	0.372830	0.069896	0.213862	0.076*	
C35	0.7429 (5)	0.3530 (4)	0.1439 (3)	0.0371 (14)	
H35	0.786706	0.413915	0.165208	0.044*	
C36	0.7979 (5)	0.3725 (5)	−0.0161 (3)	0.0453 (16)	
C37	0.6794 (6)	0.7046 (6)	0.4303 (3)	0.0557 (18)	
H37	0.609080	0.714292	0.430065	0.067*	
C38	0.5916 (6)	0.5410 (5)	0.3769 (4)	0.081 (3)	
H38A	0.600958	0.483486	0.401555	0.122*	
H38B	0.580980	0.524624	0.326648	0.122*	
H38C	0.527904	0.564064	0.383144	0.122*	
C39	0.7921 (7)	0.5879 (8)	0.4104 (6)	0.105 (3)	
H39A	0.784959	0.518092	0.413294	0.158*	
H39B	0.847203	0.622840	0.452618	0.158*	
H39C	0.814001	0.604052	0.368618	0.158*	
C40	0.9538 (5)	0.3536 (5)	0.3548 (4)	0.0463 (16)	
H40	0.935392	0.383776	0.312759	0.056*	
C41	0.9841 (6)	0.2112 (5)	0.4108 (4)	0.065 (2)	
H41A	0.954110	0.141787	0.399135	0.097*	
H41B	0.955240	0.237329	0.446703	0.097*	
H41C	1.063606	0.221560	0.428953	0.097*	
C42	0.9213 (6)	0.2051 (5)	0.2787 (4)	0.0608 (19)	
H42A	0.979681	0.172414	0.274103	0.091*	
H42B	0.907292	0.249239	0.242390	0.091*	
H42C	0.855007	0.156877	0.273144	0.091*	
C43	0.9957 (7)	0.9360 (6)	0.3131 (5)	0.082 (3)	
H43	0.990049	0.917126	0.265468	0.098*	
C44	0.9070 (10)	0.7712 (6)	0.3267 (6)	0.128 (5)	
H44A	0.843498	0.750883	0.343153	0.192*	
H44B	0.961881	0.732411	0.345527	0.192*	
H44C	0.884971	0.761842	0.275161	0.192*	
C45	0.9639 (10)	0.8940 (11)	0.4248 (5)	0.154 (6)	
H45A	0.900280	0.857548	0.435107	0.231*	
H45B	0.968865	0.963388	0.436248	0.231*	
H45C	1.029850	0.874493	0.453027	0.231*	
C46	0.3776 (6)	0.2670 (6)	0.1059 (4)	0.0589 (19)	
H46	0.418046	0.273716	0.073016	0.071*	
C47	0.3316 (8)	0.3430 (7)	0.2019 (5)	0.106 (4)	
H47A	0.345926	0.408169	0.226449	0.159*	
H47B	0.252954	0.320568	0.181171	0.159*	
H47C	0.360378	0.298846	0.235424	0.159*	
C48	0.4471 (7)	0.4373 (6)	0.1424 (4)	0.070 (2)	
H48A	0.441179	0.485984	0.177400	0.105*	
H48B	0.523450	0.431930	0.151295	0.105*	
H48C	0.419718	0.456682	0.095147	0.105*	
C49	0.0678 (8)	1.0578 (7)	0.1412 (6)	0.103 (3)	
H49A	0.142261	1.058831	0.139513	0.154*	
H49B	0.066749	1.060161	0.190227	0.154*	
H49C	0.042146	1.113894	0.120430	0.154*	
O19	−0.0023 (5)	0.9702 (5)	0.1020 (4)	0.108 (2)	
H19	0.021307	0.922150	0.118753	0.080*	
C50	0.1834 (6)	0.8513 (7)	0.1979 (5)	0.083 (3)	
H50A	0.207086	0.900443	0.238767	0.125*	
H50B	0.208845	0.877298	0.159598	0.125*	
H50C	0.214014	0.793871	0.210966	0.125*	
O20	0.0699 (5)	0.8261 (6)	0.1758 (5)	0.132 (3)	
H20	0.050600	0.766460	0.177074	0.198*	
C51A	0.069 (3)	0.2782 (11)	0.0211 (10)	0.071 (6)	0.597 (17)
H51A	0.129732	0.244935	0.024298	0.106*	0.597 (17)
H51B	0.000421	0.234140	−0.004259	0.106*	0.597 (17)
H51C	0.067257	0.298514	0.068585	0.106*	0.597 (17)
O21A	0.0821 (9)	0.3611 (7)	−0.0157 (6)	0.077 (4)	0.597 (17)
H21A	0.078070	0.343358	−0.057490	0.116*	0.597 (17)
C51B	0.043 (4)	0.2762 (16)	0.0017 (16)	0.071 (6)	0.403 (17)
H51D	0.076391	0.278281	−0.036762	0.106*	0.403 (17)
H51E	−0.036175	0.260764	−0.017945	0.106*	0.403 (17)
H51F	0.067502	0.226539	0.031400	0.106*	0.403 (17)
O21B	0.0752 (17)	0.3683 (12)	0.0429 (10)	0.098 (7)	0.403 (17)
H21B	0.100273	0.410081	0.020317	0.146*	0.403 (17)

### Atomic displacement parameters (Å^2^)

**Table:**

	*U*^11^	*U*^22^	*U*^33^	*U*^12^	*U*^13^	*U*^23^
CO1	0.0263 (4)	0.0218 (4)	0.0233 (4)	0.0016 (3)	0.0025 (3)	0.0000 (3)
CO2	0.0425 (5)	0.0503 (6)	0.0267 (4)	0.0048 (4)	0.0078 (4)	0.0030 (4)
S1	0.0314 (7)	0.0277 (8)	0.0296 (7)	0.0071 (6)	0.0003 (6)	−0.0016 (6)
S2	0.0382 (8)	0.0312 (8)	0.0276 (7)	−0.0058 (6)	0.0093 (6)	−0.0005 (6)
S3	0.0560 (10)	0.0562 (11)	0.0396 (9)	0.0177 (9)	0.0108 (8)	0.0019 (8)
S4	0.0524 (10)	0.0721 (13)	0.0316 (8)	0.0032 (9)	0.0135 (8)	0.0073 (8)
S5	0.0348 (8)	0.0428 (9)	0.0329 (8)	0.0039 (7)	−0.0001 (6)	−0.0040 (7)
S6	0.0367 (8)	0.0374 (10)	0.0634 (11)	0.0060 (7)	−0.0021 (8)	0.0088 (8)
N1	0.026 (2)	0.017 (2)	0.029 (2)	0.0034 (18)	0.0072 (19)	0.0040 (18)
N2	0.034 (2)	0.024 (2)	0.027 (2)	0.006 (2)	0.003 (2)	−0.0037 (19)
N3	0.041 (3)	0.045 (3)	0.034 (3)	0.018 (2)	0.004 (2)	−0.005 (2)
N4	0.026 (2)	0.017 (2)	0.031 (2)	0.0037 (18)	0.0035 (19)	0.0031 (19)
N5	0.030 (2)	0.019 (2)	0.031 (2)	−0.0073 (19)	0.005 (2)	−0.0014 (19)
N6	0.044 (3)	0.033 (3)	0.036 (3)	−0.008 (2)	0.014 (2)	−0.005 (2)
N7	0.052 (3)	0.048 (3)	0.032 (3)	0.009 (3)	0.013 (3)	0.006 (2)
N8	0.068 (4)	0.050 (4)	0.041 (3)	0.009 (3)	0.013 (3)	−0.005 (3)
N9	0.083 (4)	0.059 (4)	0.058 (4)	0.024 (4)	0.025 (3)	−0.003 (3)
N10	0.029 (2)	0.045 (3)	0.029 (3)	0.002 (2)	0.003 (2)	0.009 (2)
N11	0.035 (3)	0.063 (4)	0.035 (3)	−0.003 (3)	0.007 (2)	0.010 (3)
N12	0.047 (3)	0.091 (5)	0.043 (3)	−0.004 (3)	0.014 (3)	0.016 (3)
N13	0.054 (4)	0.070 (5)	0.073 (4)	0.021 (3)	0.004 (3)	−0.013 (4)
N14	0.044 (3)	0.040 (3)	0.054 (3)	0.007 (3)	0.007 (3)	0.005 (3)
N15	0.084 (5)	0.110 (7)	0.084 (6)	0.019 (5)	0.024 (5)	0.012 (5)
N16	0.055 (4)	0.070 (4)	0.057 (4)	0.027 (3)	0.024 (3)	0.021 (3)
O1	0.035 (2)	0.020 (2)	0.028 (2)	0.0018 (16)	−0.0023 (17)	−0.0023 (16)
O2	0.048 (2)	0.023 (2)	0.048 (2)	0.0123 (19)	−0.011 (2)	−0.0023 (19)
O3	0.0283 (18)	0.0203 (19)	0.0271 (19)	−0.0009 (15)	0.0064 (16)	−0.0025 (15)
O4	0.037 (2)	0.039 (2)	0.036 (2)	−0.0066 (19)	0.0124 (18)	−0.0034 (18)
O5	0.034 (2)	0.045 (3)	0.036 (2)	−0.0006 (19)	0.0032 (18)	0.0029 (19)
O6	0.032 (2)	0.057 (3)	0.064 (3)	0.005 (2)	0.001 (2)	0.015 (3)
O7	0.051 (2)	0.041 (2)	0.028 (2)	0.007 (2)	0.0118 (19)	−0.0003 (18)
O8	0.055 (3)	0.039 (3)	0.033 (2)	0.000 (2)	0.018 (2)	0.0006 (19)
O9	0.042 (2)	0.084 (4)	0.035 (2)	0.003 (2)	0.009 (2)	−0.004 (2)
O10	0.048 (3)	0.039 (3)	0.045 (3)	−0.003 (2)	−0.007 (2)	−0.015 (2)
O11	0.039 (2)	0.034 (2)	0.036 (2)	0.0010 (18)	−0.0015 (18)	0.0002 (18)
O12	0.059 (3)	0.036 (3)	0.076 (3)	0.006 (2)	0.001 (3)	0.005 (2)
O13	0.056 (3)	0.052 (3)	0.085 (4)	0.018 (2)	0.027 (3)	0.003 (3)
O14	0.049 (3)	0.057 (3)	0.070 (3)	−0.004 (2)	−0.016 (2)	0.020 (3)
O15	0.051 (3)	0.068 (4)	0.079 (4)	0.008 (3)	0.012 (3)	−0.022 (3)
O16	0.059 (3)	0.038 (3)	0.053 (3)	−0.014 (2)	0.025 (2)	−0.006 (2)
O17	0.080 (4)	0.089 (5)	0.128 (6)	0.033 (4)	−0.036 (4)	−0.007 (4)
O18	0.058 (3)	0.075 (4)	0.060 (3)	−0.001 (3)	−0.004 (3)	0.017 (3)
C1	0.029 (3)	0.024 (3)	0.028 (3)	0.001 (2)	0.004 (2)	0.000 (2)
C2	0.039 (3)	0.024 (3)	0.033 (3)	0.007 (3)	−0.002 (3)	−0.001 (2)
C3	0.039 (3)	0.029 (3)	0.037 (3)	0.009 (3)	−0.002 (3)	0.006 (3)
C4	0.040 (3)	0.034 (3)	0.032 (3)	0.003 (3)	−0.002 (3)	0.002 (3)
C5	0.033 (3)	0.026 (3)	0.030 (3)	0.001 (2)	0.003 (2)	−0.001 (2)
C6	0.025 (3)	0.027 (3)	0.027 (3)	−0.002 (2)	0.003 (2)	0.003 (2)
C7	0.055 (4)	0.026 (3)	0.057 (4)	0.014 (3)	−0.007 (3)	0.000 (3)
C8	0.033 (3)	0.019 (3)	0.024 (3)	0.002 (2)	0.004 (2)	0.001 (2)
C9	0.037 (3)	0.025 (3)	0.031 (3)	0.006 (3)	0.008 (3)	0.001 (2)
C10	0.029 (3)	0.028 (3)	0.023 (3)	0.010 (2)	0.002 (2)	0.002 (2)
C11	0.029 (3)	0.032 (3)	0.031 (3)	−0.001 (3)	0.005 (2)	0.000 (3)
C12	0.039 (3)	0.046 (4)	0.033 (3)	0.001 (3)	0.014 (3)	0.001 (3)
C13	0.054 (4)	0.043 (4)	0.036 (3)	0.002 (3)	0.018 (3)	−0.006 (3)
C14	0.044 (3)	0.033 (3)	0.034 (3)	0.002 (3)	0.008 (3)	−0.006 (3)
C15	0.034 (3)	0.026 (3)	0.027 (3)	0.004 (2)	0.005 (2)	0.004 (2)
C16	0.043 (4)	0.052 (4)	0.051 (4)	−0.011 (3)	0.019 (3)	−0.007 (3)
C17	0.027 (3)	0.024 (3)	0.026 (3)	0.002 (2)	−0.003 (2)	−0.005 (2)
C18	0.030 (3)	0.026 (3)	0.029 (3)	0.001 (2)	0.007 (2)	0.005 (2)
C19	0.038 (3)	0.057 (4)	0.033 (3)	−0.007 (3)	0.011 (3)	0.003 (3)
C20	0.038 (3)	0.054 (4)	0.042 (4)	−0.004 (3)	0.006 (3)	0.008 (3)
C21	0.037 (4)	0.075 (6)	0.056 (4)	0.000 (4)	0.009 (3)	0.012 (4)
C22	0.030 (3)	0.084 (6)	0.049 (4)	−0.006 (4)	0.004 (3)	−0.004 (4)
C23	0.053 (4)	0.069 (5)	0.041 (4)	−0.020 (4)	0.016 (3)	−0.014 (4)
C24	0.049 (4)	0.062 (5)	0.029 (3)	0.000 (4)	0.014 (3)	0.008 (3)
C25	0.039 (4)	0.064 (5)	0.074 (5)	0.006 (4)	0.001 (4)	0.012 (4)
C26	0.058 (4)	0.050 (4)	0.031 (3)	−0.006 (3)	0.016 (3)	−0.008 (3)
C27	0.074 (5)	0.052 (4)	0.045 (4)	0.019 (4)	0.027 (4)	0.009 (3)
C28	0.038 (3)	0.043 (4)	0.027 (3)	0.012 (3)	0.010 (3)	0.003 (3)
C29	0.043 (3)	0.036 (4)	0.034 (3)	0.011 (3)	0.007 (3)	0.004 (3)
C30	0.057 (4)	0.042 (4)	0.034 (3)	0.016 (3)	0.020 (3)	0.012 (3)
C31	0.060 (4)	0.046 (4)	0.034 (3)	0.009 (3)	0.014 (3)	0.001 (3)
C32	0.046 (4)	0.037 (4)	0.041 (4)	0.009 (3)	0.006 (3)	0.000 (3)
C33	0.042 (3)	0.036 (4)	0.032 (3)	0.015 (3)	0.006 (3)	0.008 (3)
C34	0.064 (4)	0.046 (4)	0.042 (4)	0.002 (3)	0.021 (3)	−0.001 (3)
C35	0.035 (3)	0.037 (4)	0.033 (3)	0.004 (3)	0.002 (3)	0.003 (3)
C36	0.029 (3)	0.059 (4)	0.042 (4)	0.003 (3)	0.003 (3)	0.013 (3)
C37	0.054 (4)	0.067 (5)	0.038 (4)	0.020 (4)	0.000 (3)	−0.009 (4)
C38	0.089 (6)	0.070 (6)	0.065 (5)	0.020 (5)	−0.008 (5)	−0.010 (4)
C39	0.079 (6)	0.113 (8)	0.127 (9)	0.035 (6)	0.033 (6)	−0.019 (7)
C40	0.039 (3)	0.046 (4)	0.052 (4)	0.003 (3)	0.013 (3)	0.005 (3)
C41	0.083 (5)	0.048 (5)	0.069 (5)	0.017 (4)	0.026 (4)	0.012 (4)
C42	0.063 (5)	0.048 (4)	0.062 (5)	0.011 (4)	0.006 (4)	−0.011 (4)
C43	0.074 (6)	0.085 (7)	0.084 (7)	0.023 (5)	0.015 (5)	0.007 (6)
C44	0.155 (11)	0.081 (8)	0.146 (10)	−0.028 (8)	0.078 (9)	−0.018 (7)
C45	0.131 (10)	0.274 (18)	0.099 (9)	0.093 (11)	0.064 (8)	0.046 (10)
C46	0.050 (4)	0.082 (6)	0.042 (4)	0.018 (4)	0.005 (3)	0.014 (4)
C47	0.133 (9)	0.106 (8)	0.131 (9)	0.060 (7)	0.098 (8)	0.035 (7)
C48	0.085 (6)	0.066 (5)	0.055 (5)	0.015 (5)	0.014 (4)	−0.002 (4)
C49	0.087 (7)	0.076 (7)	0.136 (9)	−0.008 (6)	0.034 (7)	0.008 (6)
O19	0.085 (4)	0.071 (4)	0.146 (6)	0.010 (4)	0.000 (4)	0.027 (4)
C50	0.063 (5)	0.095 (7)	0.092 (7)	0.011 (5)	0.027 (5)	0.010 (5)
O20	0.062 (4)	0.134 (7)	0.200 (8)	0.019 (4)	0.026 (5)	0.105 (7)
C51A	0.053 (16)	0.071 (7)	0.078 (12)	0.009 (6)	0.005 (13)	0.014 (8)
O21A	0.099 (8)	0.059 (7)	0.069 (8)	−0.010 (6)	0.034 (7)	0.010 (5)
C51B	0.053 (16)	0.071 (7)	0.078 (12)	0.009 (6)	0.005 (13)	0.014 (8)
O21B	0.140 (16)	0.069 (12)	0.073 (14)	0.009 (11)	0.024 (11)	0.002 (9)

### Geometric parameters (Å, º)

**Table:**

CO1—N4	1.892 (4)	C8—H8	0.9500
CO1—N1	1.893 (4)	C10—C15	1.408 (7)
CO1—O1	1.927 (4)	C10—C11	1.421 (7)
CO1—O3	1.962 (3)	C11—C12	1.376 (8)
CO1—S2	2.2247 (15)	C12—C13	1.394 (8)
CO1—S1	2.2254 (15)	C12—H12	0.9500
CO2—N10	1.897 (5)	C13—C14	1.369 (8)
CO2—N7	1.902 (5)	C13—H13	0.9500
CO2—O5	1.909 (4)	C14—C15	1.414 (8)
CO2—O7	1.954 (4)	C14—H14	0.9500
CO2—S3	2.2202 (19)	C15—C17	1.434 (7)
CO2—S4	2.2269 (17)	C16—H16A	0.9800
S1—C9	1.718 (5)	C16—H16B	0.9800
S2—C18	1.711 (5)	C16—H16C	0.9800
S3—C27	1.711 (7)	C17—H17	0.9500
S4—C36	1.710 (7)	C19—C20	1.420 (9)
S5—O9	1.437 (4)	C19—C24	1.423 (9)
S5—O10	1.442 (4)	C20—C21	1.365 (9)
S5—O11	1.453 (4)	C21—C22	1.400 (10)
S5—S6	2.123 (2)	C21—H21	0.9500
S6—O13	1.434 (5)	C22—C23	1.352 (10)
S6—O12	1.460 (5)	C22—H22	0.9500
S6—O14	1.467 (5)	C23—C24	1.420 (9)
N1—C8	1.289 (6)	C23—H23	0.9500
N1—N2	1.387 (6)	C24—C26	1.398 (9)
N2—C9	1.331 (7)	C25—H25A	0.9800
N2—H2	0.8800	C25—H25B	0.9800
N3—C9	1.323 (7)	C25—H25C	0.9800
N3—H3A	0.8800	C26—H26	0.9500
N3—H3B	0.8800	C28—C33	1.419 (8)
N4—C17	1.305 (7)	C28—C29	1.437 (8)
N4—N5	1.395 (6)	C29—C30	1.378 (8)
N5—C18	1.318 (7)	C30—C31	1.382 (9)
N5—H5A	0.8800	C30—H30	0.9500
N6—C18	1.332 (6)	C31—C32	1.379 (8)
N6—H6A	0.8800	C31—H31	0.9500
N6—H6B	0.8800	C32—C33	1.413 (8)
N7—C26	1.323 (8)	C32—H32	0.9500
N7—N8	1.376 (7)	C33—C35	1.432 (8)
N8—C27	1.340 (9)	C34—H34A	0.9800
N8—H8A	0.8800	C34—H34B	0.9800
N9—C27	1.317 (8)	C34—H34C	0.9800
N9—H9A	0.8800	C35—H35	0.9500
N9—H9B	0.8800	C37—H37	0.9500
N10—C35	1.295 (7)	C38—H38A	0.9800
N10—N11	1.391 (6)	C38—H38B	0.9800
N11—C36	1.335 (8)	C38—H38C	0.9800
N11—H11	0.8800	C39—H39A	0.9800
N12—C36	1.334 (8)	C39—H39B	0.9800
N12—H12A	0.8800	C39—H39C	0.9800
N12—H12B	0.8800	C40—H40	0.9500
N13—C37	1.353 (9)	C41—H41A	0.9800
N13—C38	1.440 (6)	C41—H41B	0.9800
N13—C39	1.470 (10)	C41—H41C	0.9800
N14—C40	1.319 (8)	C42—H42A	0.9800
N14—C41	1.448 (8)	C42—H42B	0.9800
N14—C42	1.461 (8)	C42—H42C	0.9800
N15—C43	1.337 (6)	C43—H43	0.9500
N15—C45	1.438 (6)	C44—H44A	0.9800
N15—C44	1.477 (6)	C44—H44B	0.9800
N16—C46	1.315 (9)	C44—H44C	0.9800
N16—C47	1.436 (9)	C45—H45A	0.9800
N16—C48	1.445 (9)	C45—H45B	0.9800
O1—C1	1.307 (6)	C45—H45C	0.9800
O2—C2	1.380 (6)	C46—H46	0.9500
O2—C7	1.424 (7)	C47—H47A	0.9800
O3—C10	1.317 (6)	C47—H47B	0.9800
O4—C11	1.393 (6)	C47—H47C	0.9800
O4—C16	1.433 (7)	C48—H48A	0.9800
O5—C19	1.314 (7)	C48—H48B	0.9800
O6—C20	1.378 (8)	C48—H48C	0.9800
O6—C25	1.412 (8)	C49—O19	1.441 (10)
O7—C28	1.306 (6)	C49—H49A	0.9800
O8—C29	1.373 (7)	C49—H49B	0.9800
O8—C34	1.419 (7)	C49—H49C	0.9800
O15—C37	1.215 (8)	O19—H19	0.8400
O16—C40	1.234 (7)	C50—O20	1.398 (9)
O17—C43	1.189 (6)	C50—H50A	0.9800
O18—C46	1.232 (9)	C50—H50B	0.9800
C1—C6	1.404 (7)	C50—H50C	0.9800
C1—C2	1.441 (8)	O20—H20	0.8400
C2—C3	1.373 (7)	C51A—O21A	1.427 (7)
C3—C4	1.397 (8)	C51A—H51A	0.9800
C3—H3	0.9500	C51A—H51B	0.9800
C4—C5	1.371 (8)	C51A—H51C	0.9800
C4—H4	0.9500	O21A—H21A	0.8400
C5—C6	1.430 (7)	C51B—O21B	1.428 (7)
C5—H5	0.9500	C51B—H51D	0.9800
C6—C8	1.429 (7)	C51B—H51E	0.9800
C7—H7A	0.9800	C51B—H51F	0.9800
C7—H7B	0.9800	O21B—H21B	0.8400
C7—H7C	0.9800		
			
N4—CO1—N1	175.64 (18)	H16B—C16—H16C	109.5
N4—CO1—O1	88.27 (16)	N4—C17—C15	125.0 (5)
N1—CO1—O1	94.70 (16)	N4—C17—H17	117.5
N4—CO1—O3	94.34 (16)	C15—C17—H17	117.5
N1—CO1—O3	88.98 (15)	N5—C18—N6	119.0 (5)
O1—CO1—O3	87.66 (15)	N5—C18—S2	119.5 (4)
N4—CO1—S2	87.23 (13)	N6—C18—S2	121.4 (4)
N1—CO1—S2	89.55 (13)	O5—C19—C20	117.9 (6)
O1—CO1—S2	90.33 (12)	O5—C19—C24	124.7 (6)
O3—CO1—S2	177.41 (11)	C20—C19—C24	117.4 (6)
N4—CO1—S1	89.69 (13)	C21—C20—O6	125.6 (7)
N1—CO1—S1	87.39 (13)	C21—C20—C19	121.6 (7)
O1—CO1—S1	177.78 (11)	O6—C20—C19	112.8 (5)
O3—CO1—S1	91.61 (11)	C20—C21—C22	120.5 (7)
S2—CO1—S1	90.46 (6)	C20—C21—H21	119.8
N10—CO2—N7	178.0 (2)	C22—C21—H21	119.8
N10—CO2—O5	87.05 (18)	C23—C22—C21	119.9 (6)
N7—CO2—O5	94.4 (2)	C23—C22—H22	120.0
N10—CO2—O7	94.30 (18)	C21—C22—H22	120.0
N7—CO2—O7	87.10 (18)	C22—C23—C24	121.6 (7)
O5—CO2—O7	88.64 (17)	C22—C23—H23	119.2
N10—CO2—S3	91.06 (15)	C24—C23—H23	119.2
N7—CO2—S3	87.51 (17)	C26—C24—C23	117.5 (7)
O5—CO2—S3	177.80 (13)	C26—C24—C19	123.4 (6)
O7—CO2—S3	90.38 (13)	C23—C24—C19	119.0 (7)
N10—CO2—S4	87.04 (14)	O6—C25—H25A	109.5
N7—CO2—S4	91.63 (15)	O6—C25—H25B	109.5
O5—CO2—S4	88.63 (13)	H25A—C25—H25B	109.5
O7—CO2—S4	176.89 (13)	O6—C25—H25C	109.5
S3—CO2—S4	92.41 (7)	H25A—C25—H25C	109.5
C9—S1—CO1	96.31 (19)	H25B—C25—H25C	109.5
C18—S2—CO1	96.30 (18)	N7—C26—C24	125.2 (6)
C27—S3—CO2	96.4 (3)	N7—C26—H26	117.4
C36—S4—CO2	96.9 (2)	C24—C26—H26	117.4
O9—S5—O10	115.0 (3)	N9—C27—N8	117.8 (7)
O9—S5—O11	112.7 (2)	N9—C27—S3	123.1 (6)
O10—S5—O11	113.9 (2)	N8—C27—S3	119.1 (5)
O9—S5—S6	105.1 (2)	O7—C28—C33	125.8 (5)
O10—S5—S6	105.2 (2)	O7—C28—C29	117.5 (5)
O11—S5—S6	103.40 (19)	C33—C28—C29	116.7 (5)
O13—S6—O12	114.0 (3)	O8—C29—C30	125.4 (5)
O13—S6—O14	113.2 (3)	O8—C29—C28	113.4 (5)
O12—S6—O14	114.8 (3)	C30—C29—C28	121.2 (6)
O13—S6—S5	105.7 (2)	C29—C30—C31	121.1 (6)
O12—S6—S5	105.1 (2)	C29—C30—H30	119.4
O14—S6—S5	102.4 (2)	C31—C30—H30	119.4
C8—N1—N2	116.1 (4)	C32—C31—C30	119.8 (6)
C8—N1—CO1	126.7 (4)	C32—C31—H31	120.1
N2—N1—CO1	117.0 (3)	C30—C31—H31	120.1
C9—N2—N1	119.6 (4)	C31—C32—C33	120.8 (6)
C9—N2—H2	120.2	C31—C32—H32	119.6
N1—N2—H2	120.2	C33—C32—H32	119.6
C9—N3—H3A	120.0	C32—C33—C28	120.4 (5)
C9—N3—H3B	120.0	C32—C33—C35	116.6 (5)
H3A—N3—H3B	120.0	C28—C33—C35	123.0 (5)
C17—N4—N5	115.8 (4)	O8—C34—H34A	109.5
C17—N4—CO1	126.9 (4)	O8—C34—H34B	109.5
N5—N4—CO1	117.3 (3)	H34A—C34—H34B	109.5
C18—N5—N4	118.9 (4)	O8—C34—H34C	109.5
C18—N5—H5A	120.5	H34A—C34—H34C	109.5
N4—N5—H5A	120.5	H34B—C34—H34C	109.5
C18—N6—H6A	120.0	N10—C35—C33	125.1 (6)
C18—N6—H6B	120.0	N10—C35—H35	117.4
H6A—N6—H6B	120.0	C33—C35—H35	117.4
C26—N7—N8	117.0 (5)	N12—C36—N11	118.2 (6)
C26—N7—CO2	125.7 (5)	N12—C36—S4	122.6 (5)
N8—N7—CO2	117.3 (4)	N11—C36—S4	119.2 (5)
C27—N8—N7	119.2 (6)	O15—C37—N13	125.8 (7)
C27—N8—H8A	120.4	O15—C37—H37	117.1
N7—N8—H8A	120.4	N13—C37—H37	117.1
C27—N9—H9A	120.0	N13—C38—H38A	109.5
C27—N9—H9B	120.0	N13—C38—H38B	109.5
H9A—N9—H9B	120.0	H38A—C38—H38B	109.5
C35—N10—N11	115.0 (5)	N13—C38—H38C	109.5
C35—N10—CO2	126.9 (4)	H38A—C38—H38C	109.5
N11—N10—CO2	118.0 (4)	H38B—C38—H38C	109.5
C36—N11—N10	118.4 (5)	N13—C39—H39A	109.5
C36—N11—H11	120.8	N13—C39—H39B	109.5
N10—N11—H11	120.8	H39A—C39—H39B	109.5
C36—N12—H12A	120.0	N13—C39—H39C	109.5
C36—N12—H12B	120.0	H39A—C39—H39C	109.5
H12A—N12—H12B	120.0	H39B—C39—H39C	109.5
C37—N13—C38	121.0 (6)	O16—C40—N14	125.4 (6)
C37—N13—C39	122.6 (7)	O16—C40—H40	117.3
C38—N13—C39	116.3 (7)	N14—C40—H40	117.3
C40—N14—C41	119.4 (6)	N14—C41—H41A	109.5
C40—N14—C42	120.8 (6)	N14—C41—H41B	109.5
C41—N14—C42	119.9 (6)	H41A—C41—H41B	109.5
C43—N15—C45	125.0 (9)	N14—C41—H41C	109.5
C43—N15—C44	123.6 (8)	H41A—C41—H41C	109.5
C45—N15—C44	110.6 (9)	H41B—C41—H41C	109.5
C46—N16—C47	122.4 (7)	N14—C42—H42A	109.5
C46—N16—C48	121.8 (6)	N14—C42—H42B	109.5
C47—N16—C48	115.7 (7)	H42A—C42—H42B	109.5
C1—O1—CO1	124.2 (3)	N14—C42—H42C	109.5
C2—O2—C7	116.1 (4)	H42A—C42—H42C	109.5
C10—O3—CO1	124.4 (3)	H42B—C42—H42C	109.5
C11—O4—C16	117.5 (4)	O17—C43—N15	118.2 (9)
C19—O5—CO2	126.0 (4)	O17—C43—H43	120.9
C20—O6—C25	116.2 (5)	N15—C43—H43	120.9
C28—O7—CO2	124.5 (4)	N15—C44—H44A	109.5
C29—O8—C34	116.8 (4)	N15—C44—H44B	109.5
O1—C1—C6	126.5 (5)	H44A—C44—H44B	109.5
O1—C1—C2	116.5 (5)	N15—C44—H44C	109.5
C6—C1—C2	117.1 (5)	H44A—C44—H44C	109.5
C3—C2—O2	124.6 (5)	H44B—C44—H44C	109.5
C3—C2—C1	121.6 (5)	N15—C45—H45A	109.5
O2—C2—C1	113.8 (4)	N15—C45—H45B	109.5
C2—C3—C4	120.4 (5)	H45A—C45—H45B	109.5
C2—C3—H3	119.8	N15—C45—H45C	109.5
C4—C3—H3	119.8	H45A—C45—H45C	109.5
C5—C4—C3	120.1 (5)	H45B—C45—H45C	109.5
C5—C4—H4	120.0	O18—C46—N16	125.9 (7)
C3—C4—H4	120.0	O18—C46—H46	117.0
C4—C5—C6	120.7 (5)	N16—C46—H46	117.0
C4—C5—H5	119.6	N16—C47—H47A	109.5
C6—C5—H5	119.6	N16—C47—H47B	109.5
C1—C6—C8	122.7 (5)	H47A—C47—H47B	109.5
C1—C6—C5	120.1 (5)	N16—C47—H47C	109.5
C8—C6—C5	117.1 (5)	H47A—C47—H47C	109.5
O2—C7—H7A	109.5	H47B—C47—H47C	109.5
O2—C7—H7B	109.5	N16—C48—H48A	109.5
H7A—C7—H7B	109.5	N16—C48—H48B	109.5
O2—C7—H7C	109.5	H48A—C48—H48B	109.5
H7A—C7—H7C	109.5	N16—C48—H48C	109.5
H7B—C7—H7C	109.5	H48A—C48—H48C	109.5
N1—C8—C6	125.0 (5)	H48B—C48—H48C	109.5
N1—C8—H8	117.5	O19—C49—H49A	109.5
C6—C8—H8	117.5	O19—C49—H49B	109.5
N3—C9—N2	119.4 (5)	H49A—C49—H49B	109.5
N3—C9—S1	122.1 (4)	O19—C49—H49C	109.5
N2—C9—S1	118.5 (4)	H49A—C49—H49C	109.5
O3—C10—C15	125.5 (5)	H49B—C49—H49C	109.5
O3—C10—C11	119.0 (5)	C49—O19—H19	109.5
C15—C10—C11	115.5 (5)	O20—C50—H50A	109.5
C12—C11—O4	124.3 (5)	O20—C50—H50B	109.5
C12—C11—C10	123.0 (5)	H50A—C50—H50B	109.5
O4—C11—C10	112.7 (5)	O20—C50—H50C	109.5
C11—C12—C13	119.8 (5)	H50A—C50—H50C	109.5
C11—C12—H12	120.1	H50B—C50—H50C	109.5
C13—C12—H12	120.1	C50—O20—H20	109.5
C14—C13—C12	119.8 (5)	O21A—C51A—H51A	109.5
C14—C13—H13	120.1	O21A—C51A—H51B	109.5
C12—C13—H13	120.1	H51A—C51A—H51B	109.5
C13—C14—C15	120.4 (5)	O21A—C51A—H51C	109.5
C13—C14—H14	119.8	H51A—C51A—H51C	109.5
C15—C14—H14	119.8	H51B—C51A—H51C	109.5
C10—C15—C14	121.4 (5)	C51A—O21A—H21A	109.5
C10—C15—C17	123.5 (5)	O21B—C51B—H51D	109.5
C14—C15—C17	115.1 (5)	O21B—C51B—H51E	109.5
O4—C16—H16A	109.5	H51D—C51B—H51E	109.5
O4—C16—H16B	109.5	O21B—C51B—H51F	109.5
H16A—C16—H16B	109.5	H51D—C51B—H51F	109.5
O4—C16—H16C	109.5	H51E—C51B—H51F	109.5
H16A—C16—H16C	109.5	C51B—O21B—H21B	109.5

### Hydrogen-bond geometry (Å, º)

**Table:**

*D*—H···*A*	*D*—H	H···*A*	*D*···*A*	*D*—H···*A*
N8—H8*A*···O8^i^	0.88	2.28	2.969 (7)	135
N2—H2···O3^ii^	0.88	2.27	2.999 (5)	140
N2—H2···O4^ii^	0.88	2.01	2.740 (6)	140
N11—H11···O14^iii^	0.88	2.02	2.877 (7)	165
N5—H5*A*···O11	0.88	1.98	2.813 (6)	157
N12—H12*B*···O9^iv^	0.88	2.12	2.911 (7)	150
N3—H3*B*···O17^v^	0.88	1.98	2.839 (8)	166
N3—H3*A*···O15^ii^	0.88	2.05	2.881 (7)	156
N9—H9*A*···O18^i^	0.88	1.89	2.756 (8)	168
N6—H6*B*···O16^vi^	0.88	1.95	2.822 (6)	169
N9—H9*B*···O19^vii^	0.88	1.97	2.834 (9)	165
N12—H12*A*···O21*A*^iv^	0.88	2.06	2.878 (12)	155
O19—H19···O20	0.84	1.90	2.720 (9)	167
O20—H20···O12	0.84	2.01	2.722 (8)	142

Symmetry codes: (i) −*x*+1, −*y*, −*z*; (ii) −*x*+1, −*y*+2, −*z*+1; (iii) *x*+1, *y*, *z*; (iv) −*x*+1, −*y*+1, −*z*; (v) *x*−1, *y*, *z*; (vi) −*x*+1, −*y*+1, −*z*+1; (vii) *x*+1, *y*−1, *z*.
